# Global Trends in Mortality From Ischemic Heart Disease and the Expansion of Interventional Cardiology Procedures: An Analysis Using the Global Burden of Disease (GBD) Dataset

**DOI:** 10.7759/cureus.102070

**Published:** 2026-01-22

**Authors:** Efeturi M Okorigba, Tochukwu W Okahia, Chibuzo C Manafa, Feyisayo O Oguntuase, Nonso Ariahu, Anita S Asamoah-Twum, Said R Tindwa, Ugochukwu N Ugwu

**Affiliations:** 1 Internal Medicine, West Virginia University, Morgantown, USA; 2 Internal Medicine, Leeds Teaching Hospital NHS Trust, Leeds, GBR; 3 Family Medicine, Queensland Medical Clinic, Calgary, CAN; 4 General Medicine, National Pirogov Memorial Medical University, Vinnytsia, UKR; 5 Medicine and Surgery, College of Medicine University of Lagos, Lagos, NGA; 6 General Practice, NHS Education for Scotland (Queen Elizabeth University Hospital), Glasgow, GBR; 7 Internal Medicine, Tula State University, Tula, RUS; 8 Public Health Sciences, Teesside University, Middlesbrough, GBR

**Keywords:** cardiovascular health, global burden, interventional cardiology, ischemic heart disease, mortality trends, regional disparities

## Abstract

Background: Ischemic heart disease (IHD) remains the leading cause of global mortality, with notable regional disparities reflecting differences in healthcare access, socioeconomic status, and interventional cardiology capacity.

Objective: To assess global and regional trends in ischemic heart disease (IHD) mortality from 2000 to 2021 using Global Burden of Disease (GBD) data across seven regions.

Methods: A descriptive analytical design was applied using age-standardized IHD mortality data extracted from the GBD database for the years 2000-2021. Regional averages and percentage changes were computed to examine temporal trends.

Results: A general decline in IHD mortality was observed globally, with the most pronounced reductions in Western and Eastern Europe, moderate declines in Latin America, East Asia, and globally overall, and minimal change in Sub-Saharan Africa. South Asia showed a slight increase. The variations closely align with differences in access to interventional cardiology services and preventive cardiovascular programs.

Conclusion: Despite overall global improvement, persistent disparities highlight the need to strengthen interventional cardiology capacity and preventive care systems in low- and middle-income regions to achieve equitable cardiovascular outcomes.

## Introduction

Ischemic heart disease (IHD) has been a major cause of mortality in the world, with an estimated 9 million deaths each year and close to 16% of the total worldwide deaths [1]. In the last 20 years, IHD has continued to cause a considerable burden on health and the economy, both in developed and developing countries, even with the efforts made in prevention, diagnosis, and management of the disease [2]. The mortality trends, however, show a vivid inequality between different regions of the world, which is an indicator of healthcare access, socioeconomic development, and access to interventional cardiology procedures, including percutaneous coronary intervention (PCI) and coronary artery bypass grafting (CABG) [3, 4].

Cardiovascular care in the last 20 years has been characterized by an impressive growth in interventional cardiology as one of the fields that incorporates the use of minimally invasive approaches, such as angioplasty and stenting, to repair the coronary blood flow [5]. The adoption of technologies and the secondary prevention measures such as statin treatment, hypertensive control, and smoking cessation interventions has resulted in impressive reductions in the mortality caused by IHD in high-income areas, especially Western Europe and selected parts of North America [6-7]. Other countries that have registered greater than 40% decreases in age-standardized mortality related to IHD since 2000 include the United Kingdom, France, and Germany, and this is mostly because of enhanced acute coronary treatments, extensive access to PCI, and health policies aimed at cardiovascular risk factors [8].

Low- and middle-income areas, such as Sub-Saharan Africa, South Asia, and some elements of Latin America, maintain stagnant or increasing death rates of IHD [9, 10]. All of these areas experience the intersection of epidemiological change and systemic health inequity: increased prevalence of risk factors like diabetes, hypertension, and obesity, and inadequate access to cardiac diagnostics, revascularization procedures, and secondary prevention of these conditions [11]. Likewise, the situation has not improved in South Asia, where IHD tends to manifest a decade before Western populations, where the lack of capacity to perform PCI and the absence of an effective emergency response system have prevented improvements despite greater awareness of the disease [12, 13].

Meanwhile, East Asia offers a different trend, with the fast development of industrialization and modernization of the health system [14]. Mixed trends in mortality have been observed in countries like China and South Korea, where the prevalence of IHD risk factors is on the increase, but interventional cardiology programs are increasing at the same time [15, 16]. Latin America and the Caribbean, however, have transitional trends with such countries as Brazil and Chile making progress because their universal health services have been strengthened, and networks of catheterization labs are growing [17].

The study utilized the Global Burden of Disease (GBD) dataset. The GBD data provides a strong platform to measure these changes and see where progress has been achieved or where gaps exist [18, 19]. The main objective of this study was to evaluate global and regional trends in IHD mortality from 2000 to 2021, drawing from GBD data across seven regions: Western Europe, Eastern Europe, East Asia, South Asia, Sub-Saharan Africa, Latin America and the Caribbean, and the global aggregate. Differences in trends are subsequently discussed in relation to disparities in access to interventional cardiology services. The measurement of international and regional changes in mortality due to IHD, as the expansion of interventional cardiology is instrumental in comprehending how technological changes can be achieved by real-life health data [20].

## Materials and methods

Study design and data source

This study employed a descriptive ecological design using publicly available secondary data from the GBD study 2021 estimates, accessed through the Global Health Data Exchange (GHDx) Results Tool [21]. The GBD database provides standardized and comparable estimates of cause-specific mortality derived from multiple sources, including national vital registration systems, hospital records, verbal autopsy data, and population-based surveys, which are synthesized using established statistical modeling techniques. Data were extracted for the period 2000 to 2021 to capture long-term temporal trends in IHD mortality using the GBD Results query interface with the following parameters specified: estimate type set to GBD Estimates, measure set to deaths, metric set to rate, cause to IHD, age set to age-standardized, sex set to both sexes combined, years covering 2000-2021, and locations including the global aggregate and the regions of Western Europe, Eastern Europe, East Asia, South Asia, Sub-Saharan Africa, and Latin America and the Caribbean [21]. All data were downloaded in tabular (CSV) format directly from the GHDx interface, and age-standardized mortality rates (per 100,000 population) were used to ensure comparability across regions and over time.

Study population

The analysis included seven geographical regions represented in the GBD dataset: Western Europe, Eastern Europe, East Asia, South Asia, Sub-Saharan Africa, Latin America and the Caribbean, and the global aggregate. These regions were selected to provide a balanced representation of diverse economic and healthcare contexts, particularly with respect to the availability and development of interventional cardiology services. Including both high- and low-resource settings allowed for meaningful comparison of mortality trends across different stages of cardiovascular care infrastructure development.

Variables and measures

The primary outcome variable was the age-standardized mortality rate for IHD (per 100,000 population) for each region and year between 2000 and 2021. Data were extracted for both sexes combined (“both”) to provide an overall mortality trend. Secondary measures included absolute and percentage change in IHD mortality between 2000 and 2021, computed to assess the magnitude and direction of change over time.

Statistical analysis

Data were analyzed using Stata version 18 (StataCorp, College Station, TX). Descriptive statistics were used to summarize mortality rates across regions and years. Two summary tables were generated: one presenting the mean mortality rates by year and region, and another showing the absolute and percentage changes from 2000 to 2021. A complementary bar chart was produced to illustrate the regional percentage change in IHD mortality during the study period. All analyses were conducted using aggregate-level data without weighting or imputation since the GBD estimates are already adjusted for age and population structure.

Missing data

As the study utilized fully processed and validated aggregate data from the GBD database, there were no missing values in the extracted dataset. The data completeness ensured consistency and comparability across years and regions.

Ethical consideration

Ethical approval was not required for this study because it relied exclusively on publicly available, de-identified secondary data obtained from the GHDx. No individual-level or confidential information was accessed or analyzed.

## Results

Table 1 below presents the average age-standardized mortality rate from IHD across seven global regions from 2000 to 2021. Rates represent the mean annual number of deaths per 100,000 population, as estimated by the GBD study.

**Table 1:** Mean Mortality per 100,000 Population (2000-2021)

Region	Mean IHD Mortality per 100,000 (2000-2021)
Western Europe	69.88
Sub-Saharan Africa	72.92
Latin America and Caribbean	88.54
East Asia	99.25
Global	118.87
South Asia	141.49
Eastern Europe	338.55

As shown in the findings above, it is evident that IHD mortality varied considerably across regions over the study period. Eastern Europe had the highest mean mortality rate (338.55 per 100,000), nearly three times the global average, reflecting a sustained burden despite improvements elsewhere. South Asia and the global estimate also remained above 100 per 100,000, indicating a persistent regional challenge. In contrast, Western Europe recorded the lowest average rate (69.88 per 100,000), consistent with long-standing advances in cardiovascular prevention, early diagnosis, and interventional treatment. Sub-Saharan Africa and Latin America displayed intermediate levels, suggesting gradual but uneven progress in reducing IHD mortality.

Table 2 below summarizes the temporal change in age-standardized mortality rates from IHD between 2000 and 2021 across seven global regions.

**Table 2:** Change in Age-Standardized Mortality Rate From Ischemic Heart Disease (IHD), 2000–2021 The absolute change represents the numeric difference in mortality per 100,000 population, while the percentage change reflects the relative variation over the study period. The “Trend” column provides a qualitative description of each region’s trajectory based on the direction and magnitude of change.

Region	2000	2021	Absolute Change	% Change	Trend
Western Europe	103.8	47.8	-56	-53.90%	Sharp decline
Eastern Europe	396.4	248.5	-147.9	-37.30%	Decline
Global	140.1	102.9	-37.2	-26.50%	Decline
Latin America and the Caribbean	101.7	86.5	-15.2	-15.00%	Slight decline
East Asia	102.3	93.8	-8.5	-8.30%	Mild decline
Sub-Saharan Africa	75.3	73	-2.3	-3.00%	Stable
South Asia	133.8	136.4	2.6	1.94%	Slight increase

Between 2000 and 2021, global IHD mortality demonstrated an overall downward trend, although the extent of decline varied substantially across regions. Western Europe showed the sharpest reduction (-53.9%), more than halving its mortality rate over two decades, a reflection of sustained advances in preventive care, early detection, and the widespread adoption of interventional cardiology procedures. Eastern Europe also experienced a notable decline (-37.3%), though rates remained considerably higher than in other regions, suggesting persistent gaps in healthcare infrastructure and secondary prevention.

In contrast, regions such as East Asia and Latin America and the Caribbean displayed more modest reductions of less than 15%, indicating slower progress in IHD control. Sub-Saharan Africa exhibited relative stability, with only a minor 3% reduction, highlighting ongoing challenges in access to specialized cardiac care. Uniquely, South Asia recorded a slight increase (+1.9%) in IHD mortality, signaling a growing disease burden likely linked to demographic expansion, rising risk factors, and uneven healthcare accessibility.

Overall, the data reveal that while global mortality from IHD has declined, significant disparities remain between regions. The most pronounced improvements were observed in high-income regions with well-established interventional cardiology systems, while low- and middle-income areas continue to face limited progress.

**Figure 1:**
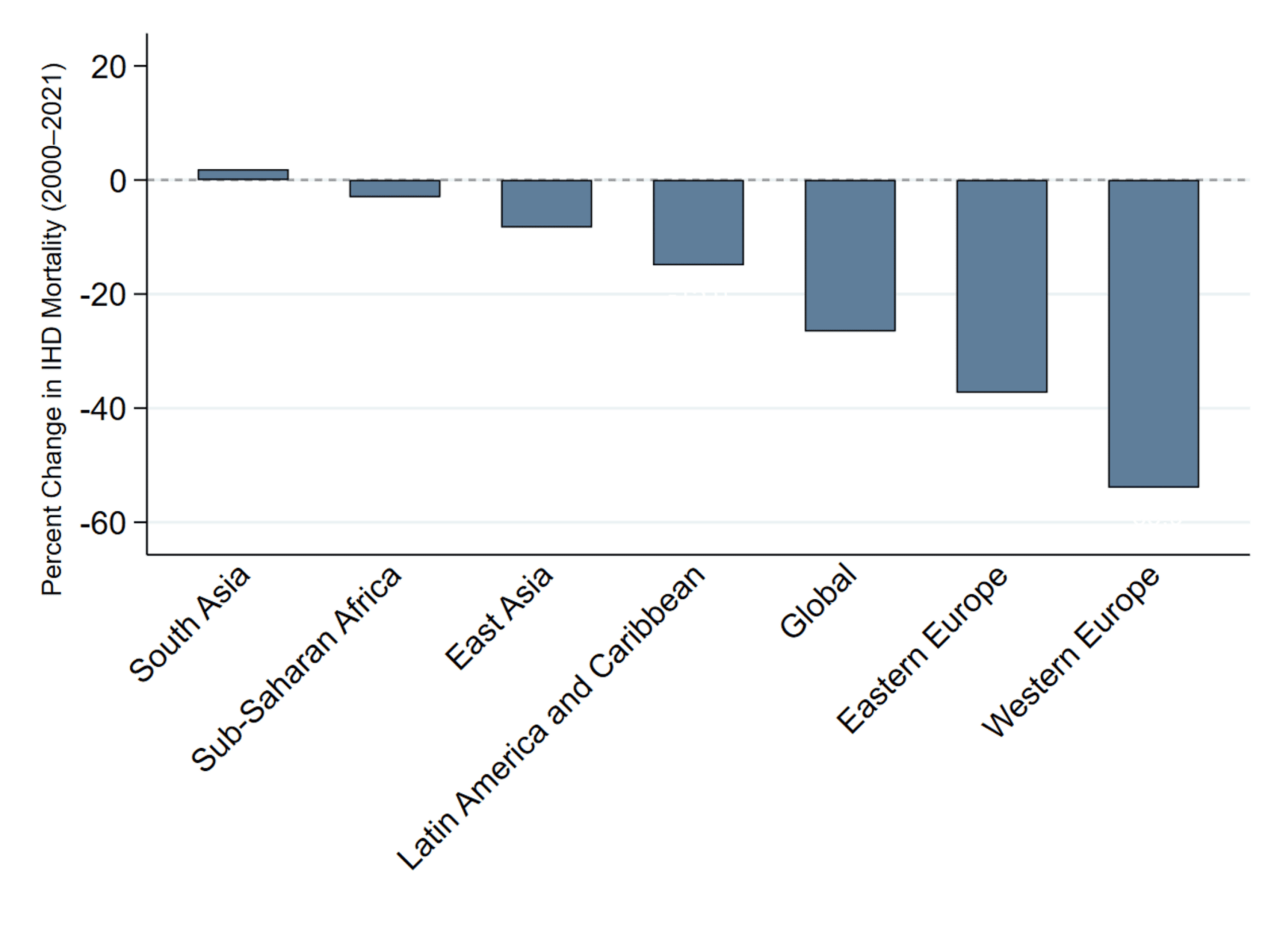
Percent Change in Ischemic Heart Disease (IHD) Mortality by Region, 2000–2021

Figure 1 illustrates the percentage change in age-standardized mortality rates from IHD between 2000 and 2021 across seven global regions.

From the figure above, it is evident that there is a regional contrast in changes in IHD mortality between 2000 and 2021. Most regions experienced a downward trend, indicating overall progress in cardiovascular health. The largest reductions were observed in high-income areas, while several low- and middle-income regions showed minimal change or slight increases. This pattern reflects ongoing disparities in access to preventive care, early detection, and interventional cardiology services across regions.

## Discussion

This study examined global and regional trends in IHD mortality between 2000 and 2021 using data from the GBD study. The analysis revealed a general decline in age-standardized IHD mortality worldwide, although the rate and direction of change varied markedly across regions. High-income areas such as Western and Eastern Europe showed substantial reductions, while Sub-Saharan Africa and South Asia exhibited minimal declines or slight increases [21]. These variations mirror differences in healthcare infrastructure, socioeconomic development, and access to interventional cardiology services.

The marked decrease in IHD mortality in Western Europe aligns with prior studies highlighting the effects of widespread implementation of primary and secondary prevention strategies, including smoking cessation, lipid control, and hypertension management [6-8]. Additionally, the expansion of interventional cardiology, particularly PCI and CABG, has played a central role in improving survival following acute coronary events [5]. The robust declines in mortality across these regions demonstrate the cumulative impact of coordinated cardiovascular policies, early detection, and access to advanced therapeutic technologies.

In contrast, Eastern Europe, though showing improvement, continues to have the highest IHD mortality rates. The slower decline may reflect persistent socioeconomic inequalities and the delayed diffusion of modern interventional cardiology practices [3, 8]. The region’s historical burden of cardiovascular risk factors, particularly smoking and high blood pressure, remains a major contributor to mortality despite health system reforms.

Low- and middle-income regions such as Sub-Saharan Africa, South Asia, and Latin America presented contrasting outcomes. Sub-Saharan Africa experienced negligible change over the 21-year period, which corresponds with evidence of limited access to diagnostic and therapeutic cardiac care [9-11]. South Asia remains a cause for concern, showing a slight upward trend despite growing awareness of cardiovascular disease. This stagnation is consistent with reports describing the early onset of IHD, limited catheterization facilities, and inadequate emergency care systems [12,13]. The persistence of high mortality in these regions underscores the widening global gap in cardiovascular outcomes.

Latin America and the Caribbean showed modest reductions in mortality, reflecting a transitional epidemiological profile. The improvements in countries such as Brazil and Chile have been attributed to expanding universal health coverage and investment in cardiac care networks [17]. Meanwhile, East Asia showed mild declines, representing a mixed pattern where industrialization and lifestyle changes elevate risk, but concurrent health system modernization mitigates adverse outcomes [14-16]. Together, these regional trends reveal that access to interventional cardiology is a critical determinant of IHD mortality progress, though its impact is moderated by broader health system capacity and preventive efforts.

The findings reinforce that technological advancement alone cannot reduce IHD mortality globally. Broader strategies integrating primary prevention, equitable resource distribution, and regional capacity building are needed to sustain progress. Countries lagging behind could benefit from tailored investments in cardiac diagnostics, PCI infrastructure, and post-event rehabilitation programs.

Implications for global health policy and strengthening of systems

Population-based prevention programs should be grounded in the World Health Organization (WHO) “Best Buys,” which are cost-effective, evidence-based strategies designed to reduce the burden of non-communicable diseases, including IHD [22,23]. These interventions include comprehensive tobacco control policies, reduction of salt content in processed foods, elimination of industrial trans-fats, and promotion of physical activity through the creation of safe and accessible public spaces. Additional measures include cancer prevention strategies such as human papillomavirus (HPV) and hepatitis B vaccination, population-based screening programs for cervical, breast, and colorectal cancers, public education campaigns targeting major cardiovascular risk factors, and the treatment of common NCDs. Integrating these initiatives within schools and workplaces is essential to promote early adoption and sustained behavioral change at the societal level.

In parallel, the WHO HEARTS technical package should be integrated into primary care systems to strengthen early diagnosis and long-term control of major cardiovascular risk factors, including hypertension, diabetes mellitus, and hypercholesterolemia [24,25]. HEARTS is a standardized six-component framework supported by an implementation guide that assists ministries of health in improving cardiovascular disease management within primary care settings, thereby enhancing secondary prevention and continuity of care [24-25].

Expansion of interventional cardiology capacity is also critical. This includes increasing the number of PCI laboratories globally and establishing well-coordinated regional networks with clearly defined protocols for rapid and safe inter-facility patient transfer. PCI centers must be adequately staffed and resourced to provide continuous 24-hour coverage to ensure timely reperfusion therapy for acute coronary syndromes.

Furthermore, strengthening emergency medical services (EMS) is essential to reducing treatment delays. Centralized EMS dispatch systems capable of performing pre-hospital electrocardiograms and transmitting them directly to PCI centers enable early diagnosis and immediate cath lab activation. In settings where timely primary PCI is not feasible, pharmaco-invasive strategies consisting of early fibrinolysis followed by transfer for definitive coronary intervention should be systematically implemented [26].

Finally, health systems worldwide can improve long-term secondary prevention by implementing simple, standardized treatment protocols in primary care, particularly through the HEARTS approach. Such protocols promote medication adherence, improve risk-factor control, and enhance continuity of care, ultimately contributing to sustained reductions in IHD morbidity and mortality [24].

Strengths and limitations

A key strength of this study lies in its use of the GBD dataset, which provides standardized, comprehensive estimates across countries and years, enabling reliable comparison of mortality patterns. The inclusion of diverse regions allows for a balanced global perspective. However, as an ecological analysis based on aggregate data, the study cannot account for within-country disparities or causal relationships between interventions and outcomes. Additionally, regional averages may mask heterogeneity across nations within the same region.

Future studies should explore country-level analyses to identify contextual drivers of change, such as policy implementation, healthcare financing, and workforce capacity. Integrating GBD estimates with local clinical registries could enhance understanding of how interventional cardiology expansion translates to survival gains. Furthermore, longitudinal studies assessing access, quality, and outcomes of cardiac interventions would help inform equitable global cardiovascular care planning.

## Conclusions

This study reveals substantial global reductions in IHD mortality from 2000 to 2021, with the steepest declines observed in regions that have expanded access to interventional cardiology and preventive cardiovascular care. Nonetheless, wide regional disparities persist, with slower progress or stable trends in parts of Sub-Saharan Africa and South Asia. These findings emphasize the ongoing inequality in cardiovascular health outcomes and highlight the need to strengthen cardiac care infrastructure, preventive programs, and equitable access to interventional services worldwide.
